# 表皮生长因子/HER2抑制剂在非小细胞肺癌患者治疗中的作用综述

**DOI:** 10.3779/j.issn.1009-3419.2010.04.18

**Published:** 2010-04-20

**Authors:** Ramaswamy GOVINDAN, 娟 南, 燕 丁

**Affiliations:** 1 Alvin J. Siteman Cancer Center, Washington University School of Medicine, St Louis, MO; 2 天津医科大学总医院，天津市肺癌研究所，天津市肺癌转移与肿瘤微环境重点实验室

**Keywords:** BIBW 2992, ErbB, 厄洛替尼, 吉非替尼, HKI-272, 受体酪氨酸激酶

## Abstract

晚期非小细胞肺癌（non-small cell lung cancer, NSCLC）仍然是主要的全球健康问题。尽管可逆性表皮生长因子受体（epidermal growth factor receptor, EGFR）酪氨酸激酶抑制剂厄洛替尼可改善复发与再发NSCLC患者的生存期，但也存在明显的局限性，包括仅对少数患者亚群具有临床疗效、生存率较低及产生耐药性。EGFR和HER2的非可逆性抑制剂是临床开发的新型药物，有可能预防并克服第一代EGFR抑制剂的获得性耐药。

## 前言

非小细胞肺癌(non-small cell lung cancer, NSCLC)——最常见的肺癌类型，是美国乃至全世界癌症死亡的首位原因^[1, 2]^。多数患者呈现局部晚期肺癌或转移性肺癌，并采用基于铂类的联合化疗来治疗；但是，与最佳支持治疗相比，此类治疗的有效率(response rates, RRs)较低，且总生存期(overall survival, OS)改善甚微。基于ErbB受体家族在NSCLC和其它人类恶性肿瘤的生长和转移中所起的关键作用，表皮生长因子受体(epidermal growth factor receptor, EGFR)酪氨酸激酶抑制剂(tyrosine kinase inhibitors, TKIs)已发展成为靶向抗肿瘤药物。

目前，在美国和世界各地，小分子EGFR TKI厄洛替尼已被批准用于晚期NSCLC患者的二、三线治疗。厄洛替尼获得监管部门的批准基于Ⅲ期BR.21试验的结果，此结果显示，与安慰剂相比，厄洛替尼可给患者带来生存期获益^[3]^。尽管另一EGFR TKI吉非替尼在美国最初获得监管部门的批准，但是Ⅲ期ISEL(Iressa Survival Evaluation in Lung Cancer)显示其与安慰剂相比无生存期获益，随后其指征被仅限于曾获益于吉非替尼治疗的患者^[4]^。厄洛替尼与吉非替尼均为EG F R TK区三磷酸腺苷(adenosine triphosphate, ATP)结合位点的可逆性竞争性抑制剂。仅少数NSCLC患者采用EGFR TKI治疗有效(约10%的白种人和30%-40%东亚患者)。有研究发现，有效性与特定的分子特征相关^[5]^，特别是EGFR活化突变^[6]^。还有研究显示，EGFR基因拷贝数的升高与EGFR TKI的有效性相关^[7-9]^。尽管可逆性EGFR TKI具有诸多优点，但是在多数起初有效的患者中，这些药物的疗效受限于耐药性的产生，这将导致在中位时间12个月之后患者出现肿瘤进展和肿瘤复发^[10]^。

现有治疗的局限性、NSCLC的高发病率和晚期肺癌患者的高死亡率促使人们探寻新型药物。HER2为ErbB受体家族的另一成员，对EGFR和HER2具有抑制作用的化合物是一类处于临床研发阶段的针对晚期NSCLC患者新型药物。在此，我们将对采用EGFR/HER2抑制剂作为抗癌药物的科学原理进行综述，并将对用于治疗NSCLC患者的这些药物的临床研发做一概述。

## 非小细胞肺癌中ErbB/ HER受体的作用

从结构上来看，表皮生长因子受体是4个HER家族相关受体之一。HER家族的每一成员均由胞外生长因子结合区、单一跨膜区、胞内TK区和含有可能发生磷酸化的酪氨酸残基的胞质尾区组成(图 1)。然而，HER2无已知配体，HER3无激酶活性。这些受体通过一系列复杂的第二信使起作用，可影响各种细胞功能，包括凋亡、迁移、生长、粘附和分化。现有多种刺激性配体，包括EGF、转化生长因子(transforming growth factor, TGF) -α和神经调节蛋白，这些刺激性配体对不同的HER家族成员呈现不同的特性(图 1) ^[11]^。同源配体和受体的类型和数量决定着受体激活引发的生物反应。

**1:**
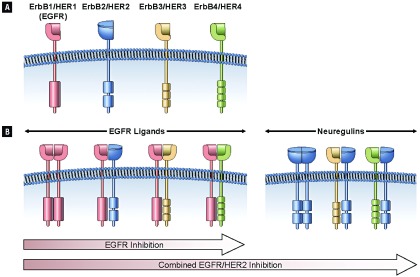
ErbB信号的协同作用和所有EGFR抑制剂对比EGFR/HER2抑制剂的不同活性 Cooperative ErbB Signaling and Differential Activity of Sole EGFR Inhibition Versus EGFR/HER2 Inhibition

NSCLC细胞的信号传导有赖于多种HER家族成员的共表达与协同作用。配体结合所触发的受体同源二聚化和异源二聚化是EGFR/HER信号传导的必经步骤。二聚化可触发受体内在TK活性的活化，继而可引起各种第二信使的募集。HER家族成员间的相互作用可影响配体结合后生物反应的类型和持续时间。

禽流红细胞增多症病毒的产物v-ErbB致癌基因是EGFR衍生的有活性的变异体，这一发现首次证实EGFR和其它HER家族受体与癌症相关^[12]^。EGFR通路的过度活化可引起各种人类恶性肿瘤的发生和进展。有研究表明EGFR突变具有致癌性：L858R和G719S的替代突变、外显子19的缺失突变和外显子20的插入突变可引起配体依赖性细胞转化^[13]^。编码TK区的外显子21中的L858R活化突变是最常见的NSCLC突变。EGFR活化与肿瘤细胞增殖和侵袭的增多及凋亡和化疗耐受相关^[14]^。EGFR的过表达亦见于大多数实体瘤中，包括NSCLC。这是重度吸烟者支气管上皮所见的早期异常之一，且几乎见于所有鳞癌及≥65%的大细胞癌和腺癌中^[15]^。

尽管较EGFR过表达少见，但HER2过表达亦见于NSCLC中，而且相比其它NSCLC类型(如鳞癌或大细胞癌)，在腺癌中更为多见^[16]^。在NSCLC患者中，EGFR与HER2的共过表达与临床预后不良相关^[17]^。

## EGFR/HER2抑制剂的原理

EGFR/HER2抑制剂作为抗癌治疗的科学原理源于EGFR与HER2各种分子间的相互作用，EGFR与HER2可调节HER家族信号传导通路，并使之多样化。EGFR与HER2共表达可使EGFR的有丝分裂信号被放大^[18]^。HER家族成员间的相互作用可影响配体与受体的亲和力，并可促进受体活化。例如，人类上皮细胞系统中HER2的扩增可导致HER2的组成性活化及EGFR的配体依赖性激活^[19]^。在这些研究中，HER2的扩增对EGFR信号具有长久的刺激作用，其发生经由减少EGFR的下调、降低溶酶体靶向作用、促进活化的EGFR再循环至细胞表面以及降低配体与EGFR的解离。EGFR与HER2间的上述各种相互作用导致生物信号协同作用。

EGFR与HER2间的协同作用在NSCLC的发生和进展中起关键作用。一系列临床前研究表明，*EGFR*与*HER2*基因具有使细胞转化为恶性表型的能力^[20]^。更重要的是，*EGFR*与*HER2*基因呈现协同转化潜能^[21]^。高度同步的EGFR与HER2的mRNA共表达与Ⅰ-ⅢA期NSCLC患者的不良预后相关^[17]^，随后这一现象在蛋白水平得以证实^[22]^。有假说认为同步的过表达使EGFR与HER2异源二聚化，从而导致肿瘤生长加快以及OS与无进展生存期(progression-free survival, PFS)缩短。其它研究显示，NSCLC的转移潜能与EGFR/HER2的共表达相关^[23]^。值得注意的是，在EGFR阳性(采用免疫组化或荧光原位杂交进行检测)的肿瘤中，*HER2*基因拷贝数的增多与对吉非替尼的敏感性相关，且临床疗效优于这两种受体均为阴性的肿瘤患者。这些资料为同步靶向作用于这两种受体提供了更深层次的理由^[24]^。

人们已开发出两种类型的EGFR/HER2 TKI：与TK区ATP结合位点可逆性结合的药物和与TK区ATP结合位点非可逆性(共价键)结合的药物。非可逆性EGFR/ HER2 TKI可抑制含激活突变及其它对可逆性EGFR/ HER2 TKI厄洛替尼和吉非替尼耐药突变的NSCLC细胞的活性^[25]^。非可逆性TKI克服可逆性TKI耐药突变的活性很可能归因于这些药物与EGFR TK区的共价结合^[26, 27]^。此外，与可逆性EGFR抑制剂相比，在细胞培养模型中非可逆性EGFR抑制剂的耐药性似乎较为罕见，这意味着非可逆性抑制剂可能在预防和克服耐药中均具有临床价值^[25]^。

在临床前研究中，非可逆性EGFR/HER2 TKI BIBW 2992和HKI-272(neratinib)及非可逆性EGFR抑制剂HKI-357，可干扰EGFR的自身磷酸化，并抑制对厄洛替尼和吉非替尼耐受的NSCLC细胞的生长，包括含有获得性EGFR T790M耐受突变的细胞和以T790M非依赖性耐受机制为特征的细胞，T790M非依赖性耐受机制包括受体运输的改变^[25, 28]^。BIBW 2992可抑制野生型HER2和野生型及突变型EGFR的离体TK活性，包括对厄洛替尼耐受的各种EGFR亚型。此外，BIBW 2992可抑制完整细胞中EGFR和HER2的自身磷酸化^[28]^。在体外，对厄洛替尼或吉非替尼获得性耐药且明确为EGFR L858R/T790M双重突变的NSCLC患者中，BIBW 2992的有效性约为厄洛替尼的上百倍。最近的报道显示，BIBW 2992对新的二次突变T854A有效，此突变与对可逆性EGFR TKI的获得性耐药相关。可逆性EGFR/HER2 TKI BMS-599626高度选择性地抑制依赖于EGFR/HER2的肿瘤细胞的增殖，而且受体的免疫共沉淀研究显示，这一药物可抑制经由EGFR/HER2异源二聚化的信号^[29]^。在采用12个EGFR突变(代表 5种突变类型)进行肺癌细胞系和BaF3细胞转化的研究中，HKI-272在抑制含外显子18和20突变的细胞方面较厄洛替尼更有效。相反，厄洛替尼在抑制主要为外显子19缺失突变的细胞方面较HKI-272有效^[30]^。根据后者的结果，有假说认为通过基于特定突变选择最适宜的药物可能会优化EGFR TKI的预期临床反应。BIBW 2992和HKI-272对含有HER2突变776 insV且对厄洛替尼耐受的NSCLC细胞系均有效^[28, 31]^。

非可逆性EGFR/HER2抑制剂在体内亦有效。在长时间表达2种人类EGFR突变后会发生肺腺癌的双转基因小鼠中，HKI-272具有强大的肿瘤抑制作用^[32]^。相似的是，在EGFR L585R/T790M或HER2过表达诱发的异种移植模型及EGFR L585R/T790M诱发的对厄洛替尼耐受的鼠科动物肺癌模型中，BIBW 2992具有强大的肿瘤抑制作用^[28]^。H1975 NSCLC肿瘤含有EGFR L585R/T790M突变且对厄洛替尼和吉非替尼耐受，非可逆性EGFR/HER2抑制剂AV-412可干扰H1975 NSCLC肿瘤的生长，并抑制EGFR和HER2过表达的肿瘤的生长^[33]^。AV-412以产生抗肿瘤效应的浓度可抑制EGFR和HER2的自身磷酸化。

## 临床试验

目前，数个EGFR/HER2抑制剂处于NSCLC临床研发的不同阶段(表 1)。已有的临床数据显示，此类药物通常是安全的且耐受性好，毒性谱与其它EGFR抑制剂一致。在晚期恶性肿瘤患者的早期试验中，采用各种EGFR/HER2抑制剂后会出现疾病稳定(stable disease, SD)，有时SD延长^[34-37]^。

**1:** 处于NSCLC临床研发中的EGFR/HER2抑制剂

药物/制剂	ErbB靶标	试验阶段
	非可逆性抑制剂	
BIBW2992	EGFR/HER2	Ⅲ
HKI-272 (Neratinib)	EGFR/HER2	Ⅱ
CI-1033 (Canertinib)	Pan-ErbB	不再进行NSCLC的临床研发^a^
XL647	EGFR/HER2	Ⅱ
EKB-569 (Pelitinib)	EGFR/HER2/ HER4	Ⅱ
PF-00299804	Pan-ErbB	Ⅱ
AV-412/MP-412	EGFR/HER2	Ⅰ
	可逆性抑制剂	
Lapatinib	EGFR/HER2	不再进行NSCLC的临床研发^a^
AEE788	EGFR/HER2	Ⅰ/Ⅱ
BMS-599626	Pan-ErbB	Ⅰ
Note: Reprinted with permission from the copyright holder © CIG Media Group, L.P. ^a^在Ⅱ期试验中这些化合物未达到其主要终点。其作为NSCLC单一疗法的深层次临床研发不再继续。 缩写：EGFR=表皮生长因子受体；NSCLC=非小细胞肺癌 注：本表得到版权所有者©CIG Media Group, L.P.复制许可		

## 可逆性酪氨酸激酶抑制剂

在采用可逆性TKI拉帕替尼治疗乳腺癌中，EGFR/ HER2抑制剂的临床价值得到证实。美国食品与药品监督管理局批准该药与卡培他滨联用以治疗HER2过表达的晚期或转移性乳腺癌患者。然而，由于拉帕替尼在一项Ⅱ期试验中未达到主要终点，因此对其不再进行NSCLC单一疗法的临床研发^[38]^。

## 非可逆性酪氨酸激酶抑制剂

在BIBW 2992治疗一系列实体瘤患者(*n*=26)的一项Ⅰ期试验中，初步实验结果显示肺腺癌女性患者中有2例达部分缓解(partial responses, PRs)，其中1例含有复合杂合子EGFR突变^[39]^。BIBW 2992用于治疗EGFR突变阳性且未接受过化疗或曾接受1次化疗的晚期肺腺癌患者的单臂Ⅱ期试验(LUX Lung 2)的中期结果最近得以报道。在接受二线治疗的67例可评估的患者中，43例达PS (64%; 95%CI: 52%-76%)，疾病控制率(disease control rate, DCR)为96%(95%CI: 87%-99%)，中位PFS为10.2个月(95%CI: 7.5-17.7) ^[40]^。在可评估有效性的38例未接受过化疗的患者中，RR为63%，DCR为97%^[41]^。在曾接受化疗失败的NSCLC患者的一项探索性Ⅱ期研究中，3例含有HER2突变的患者采用BIBW 2992治疗后均达客观有效，HER2突变大约见于2%-4%的腺癌患者中^[42]^。在BIBW 2992的临床试验中最常发生的不良事件(adverse events, AEs)为皮肤毒性和腹泻^[39-41]^。当前，ⅡB/Ⅲ期LUX Lung-1试验正在评估BIBW 2992克服可逆性EGFR抑制剂获得性耐药的潜能(图 2) ^[43, 44]^。旨在评估BIBW 2992 vs顺铂/培美曲塞作为含有EGFR突变的腺癌患者的一线治疗疗效的一项随机Ⅲ期试验(LUX Lung 3)已于2009年8月开始进行^[45]^。

**2:**
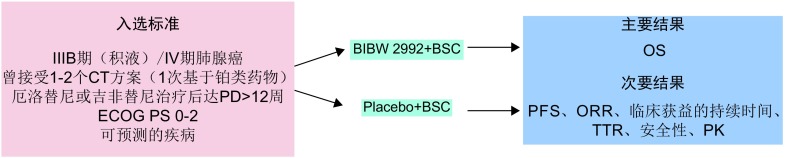
LUX Lung-1 ⅡB/Ⅲ期试验设计^[34]^ The Phase ⅡB/Ⅲ LUX Lung-1 Trial Design^[34]^

在有关HKI-272的一项Ⅰ期试验中，16例曾接受厄洛替尼或吉非替尼治疗且表达EGFR或HER2的NSCLC患者中42%达SD^[46]^。有一项Ⅱ期试验，在NSCLC患者的3个亚组中对HKI-272进行评估，这3个亚组分为：曾接受吉非替尼或厄洛替尼治疗失败且含有EGFR突变的患者(*n*=91)；突变阴性的患者(*n*=48)；未曾接受EGFR TKI治疗的患者(*n*=28)。3组间有效率、SD率和PFS无明显不同(RR，2%-4%；SD率，39%-47%；中位PFS，7.4-11.6周) ^[47]^。在HKI-272的临床试验中，腹泻为最常见的AE^[46, 47]^。

PF-00299804为非可逆性pan-HER(对EGFR、HER2和HER4具有活性) TKI，在PF-00299804的一项Ⅰ期研究中，2例达PR，在29例可评估的晚期NSCLC中，8例达SD^[48]^。一项Ⅱ期试验正在评估PF-00299804对曾接受1-2个化疗方案和厄洛替尼治疗失败的晚期NSCLC患者(KRAS为野生型)的疗效。在36例可评估的患者的初步分析中，3例达PR，临床获益率(CR+PR+SD超过2个周期，比如6周)为67%。含有T790M突变的患者的SD延长^[49]^。PF-00299804最常见的AE为皮肤疾病和胃肠疾病^[48, 49]^。

在日本患者中进行的有关非可逆性EGFR/HER2抑制剂EKB-569(pelitinib)的一项Ⅰ期试验中，含有EGFR突变并对吉非替尼获得性耐药的2例NSCLC患者均出现放射反应。最常见的AE为腹泻、皮疹、厌食和皮肤干燥^[37]^。目前，一项有关EKB-569治疗晚期NSCLC患者的Ⅱ期研究正在进行中。

## 总结

ErbB靶向药物批准用于NSCLC和其它恶性肿瘤，这表明此受体家族是抗癌治疗的有效靶标。第一代EGFR TKI为晚期NSCLC患者带来显著的临床获益，但疗效有限。克服这些局限性的最重要的改进策略是干扰HER家族成员间的协同作用，它们之间的相互作用对其生物活性至关重要。目前，非可逆性EGR/HER2抑制剂正在临床研发中，可能有助于预防和克服第一代EGFR抑制剂的获得性耐药。有关这些药物的在研Ⅲ期随机临床试验结果值得我们翘首以待。

## Acknowledgments

This work was supported by Boehringer-Ingelheim Pharmaceu-ticals, Inc. Writing and editorial assistance was provided by Johna-than Maher, PhD, of BlueSpark Healthcare Communications, and was contracted by Boehringer-Ingelheim Pharmaceuticals, Inc. Dr. Govindan meets the criteria for authorship as recommended by the International Committee of Medical Journal Editors (ICMJE), was fully responsible for all content and editorial decisions, and was involved in all stages of manuscript development. Dr. Govindan received no compensation related to the development of this article.

## Disclosures

Ramaswamy Govindan has served as a paid consultant or been on the Advisory Board of AstraZeneca; Avantis Medical Systems, Inc.; Bristol-Myers Squibb Company, Boehringer Ingelheim GmbH; and Eli Lilly and Company, and is a member of the Speaker’s Bureau of Eli Lilly and Company and Genentech, Inc.
